# Structure zone diagram and particle incorporation of nickel brush plated composite coatings

**DOI:** 10.1038/srep44561

**Published:** 2017-03-16

**Authors:** L. Isern, S. Impey, H. Almond, S. J. Clouser, J. L. Endrino

**Affiliations:** 1 School of Aerospace, Transport and Manufacturing (SATM), Cranfield University, College Road, MK43 0AL Cranfield, Bedfordshire, United Kingdom; 2 SIFCO ASC, 5708 E Schaaf Road, Independence, Ohio 44131, USA

## Abstract

This work studies the deposition of aluminium-incorporated nickel coatings by brush electroplating, focusing on the electroplating setup and processing parameters. The setup was optimised in order to increase the volume of particle incorporation. The optimised design focused on increasing the plating solution flow to avoid sedimentation, and as a result the particle transport experienced a three-fold increase when compared with the traditional setup. The influence of bath load, current density and the brush material used was investigated. Both current density and brush material have a significant impact on the morphology and composition of the coatings. Higher current densities and non-abrasive brushes produce rough, particle-rich samples. Different combinations of these two parameters influence the surface characteristics differently, as illustrated in a Structure Zone Diagram. Finally, surfaces featuring crevices and peaks incorporate between 3.5 and 20 times more particles than smoother coatings. The presence of such features has been quantified using average surface roughness Ra and Abbott-Firestone curves. The combination of optimised setup and rough surface increased the particle content of the composite to 28 at.%.

In the last two decades, Metal Matrix Composite (MMC) coatings have attracted special interest because their properties can be tailored to fit a wide range of applications. MMC coatings are generally manufactured using electroplating or electroless procedures as these technologies are simple, inexpensive and widely-available. During the process, metal ions are deposited from a liquid solution. If solid particles are present in this solution, they will be trapped by and incorporated in the depositing metal, forming a composite^1^,^2^. Since the presence of the particles can reinforce or modify certain aspects of the metal matrix^1^, choosing the right combination for the MMC has potential to enhance wear resistance^3^,^4^,^5^,^6^, corrosion protection^6^,^7^,^8^ and hardness^3^ or to reduce friction^3^,^4^,^5^, produce hydrophobic surfaces^7^ and decrease impedance^9^. As in the rule of mixtures, the final properties of the coating depend on the proportion of both matrix and particles, so the desired properties obtained depend on the amount of particles present in the coating.

MMC coatings can be produced with any electroplating technique. One such technique is brush plating, which does not require a tank to hold the solution. Instead, it uses a cloth, soaked in solution (called the brush) which distributes the electrolyte to both anode and cathode. Therefore, unlike tank electroplating or electroless plating, brush plating is compact and portable, allowing for on-site repair and plating of assembled parts. Additionally, in comparison with traditional electroplating techniques, deposition rates with brush plating are higher (usually >×10), coatings obtained are usually harder, and a smaller amount of solution is used. Further, as brush plating is localised, the amount of masking required for selective plating is smaller than with tank electroplating. Because of these advantages, brush plating is widely used for on-site repairs, coating specific areas of large parts (selective plating) and fast plating of small and medium components^10^,^11^,^12^.

MMC coating studies using brush plating have been reported from the mid-1990s^13^ but especially during the last 10 years. Some studies focus on particular characteristics that differentiate brush plating from other plating techniques, as often the information cannot be extrapolated from the more extended literature concerning tank or electroless plating. One such characteristic is the brush. It is common knowledge in the industry that using different cloth materials produces different results; nevertheless, to the best of our knowledge, only the influence of the length of the fibres has been reported until now^14^.

Another characteristic of brush plating is the use of a setup to pump the solution to the brush, collect and recirculate any excess. Traditionally, brush plating setups do not account for the presence of particles, so they are not optimised for particle transportation. In order to improve particle transport, studies of solid-liquid two-phase flow should be considered.

The present study considers solid-liquid two-phase flow principles to optimise the brush plating setup to increase particle transportation. This has been done by eliminating areas and elements that reduce the velocity of the liquid flow and by introducing agitation. The present study also analyses the influence of key deposition parameters on the particle concentration and structure of brush-plated Ni/Al coatings. Focusing on characteristics that distinguish brush plating from other plating processes, the following key parameters have been studied: the material used as brush, testing abrasive and non-abrasive cloths; the current density, testing a range of values higher than in tank plating; and the bath load, i.e. the amount of particles added into the plating solution. The composite system selected is a Ni matrix with Al particles, which has previously showed potential in corrosion protection of the Ni substrate^8^ and used as an intermediate step in the production of the intermetallic NiAl^15^.

## Experimental Methods

### Setup design

Two different plating setups were tested:

1. Traditional liquid-based setup
2. Particle-based setup.

**Figure 1:**
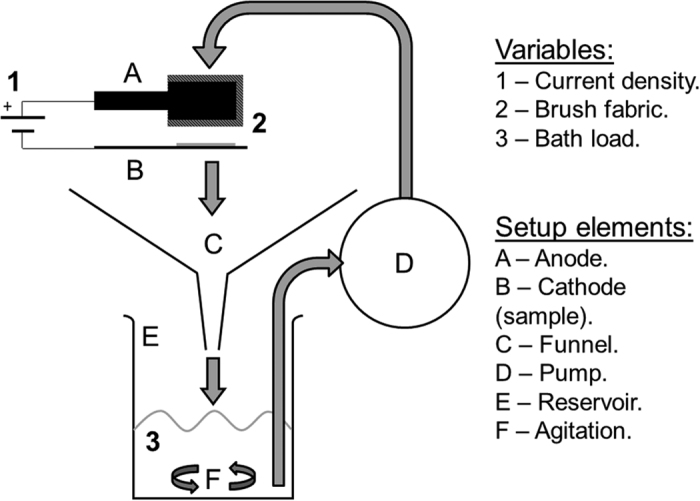
Diagram of the plating setup designed to circulate particles. The traditional design lacks the funnel (C) and the agitation (F), and the reservoir (E) is usually a shallow tray.

The traditional liquid-based setup consists of a flat tray that collects and recirculates excess liquid with a tube attached at the base connected to a peristaltic pump. In the particle-based design, solution excess is collected by a steep-walled funnel and stored in a reservoir, where magnetic stirring prevents particle sedimentation. From the reservoir, the solution is recirculated to the anode using a peristaltic pump. A schematic diagram of both setups is given in Fig. 1.

The particle-based setup has been designed following studies of solid-liquid two-phase flow. Peker and Helvaci^16^ show that particles are affected by a number of different forces, and the gravitational force is responsible for sedimentation. To avoid sedimentation, other forces should be used to balance gravity, for instance, buoyancy, drag or Brownian motion. In this case, neither Brownian, interactive nor buoyancy forces were relevant and the implemented measures aim to increase drag on the particles to minimise sedimentation. Additionally, particle velocity can show small variations with respect to liquid velocity, which complicates the design. This can be checked by calculating the Stokes number (St), which is the ratio of the particle response time to the characteristic time of the liquid, calculated using equation (1) (adapted from^16^).


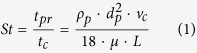


Where t_pr_ is the time response of the particle, t_c_ the time response of the continuous phase (liquid), ρ_p_ is the density of the particles, d_p_ the diameter of the particles, v_c_ the superficial velocity of the medium on the free cross-sectional area, μ the viscosity of the medium, and L the characteristic length of the medium. If St > 10, this variation in velocity can affect the particle flow, but if St < 0.1 it can be considered that the particle velocity matches the liquid velocity. In this study, St ≈ 10^−9^, therefore it is assumed that the particle velocity is comparable to the velocity of the medium and further considerations are not necessary.

For the performance test, a suspension of aluminium particles in deionized water was circulated for 30 minutes. Samples of the suspension were taken from position A in Fig. 1 at the beginning of the process (minute 1) and at the end (minute 30). The suspension samples had a known volume and were weighed to calculate the fraction of particles.

### Brush plating conditions

The SIFCO Process^®^ was followed for depositing a nickel metallic matrix on a substrate of low carbon steel (designation S275JR defined in standard BS EN 10025-2: 2011). Brush plating was undertaken with a particle-based setup with a stirring speed of 350 rpm. The appropriate solutions were used for cleaning and activating the surface. A thin layer (2 μm) of nickel was deposited by brush plating in the pre-plating step and the main plating was carried out with a nickel sulphate bath that contained aluminium particles. All solutions are industrial products manufactured by SIFCO ASC (US) and are listed in Table 1.

Brush plating was performed on an area of 30 × 30 mm, masking the remaining substrate with electroplating-grade tape. The total current applied was measured with the rectifier’s Ampere-hour counter and was set to 0.9 A·h (3240 C), which would correspond to a thickness of 80 μm if using the Nickel High Speed solution under optimal conditions without particles. The Al powder was supplied by Aluminium Powder Company Ltd (UK), with an average particle diameter of 5 μm.

**Figure 2:**
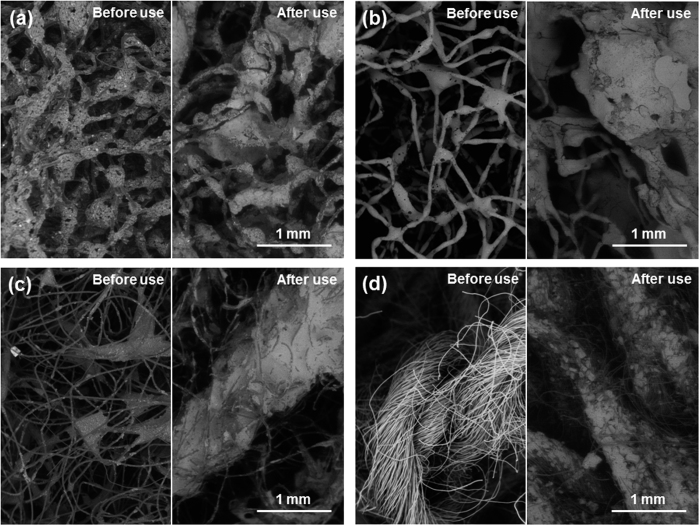
Backscattered ESEM images of the four different fabrics used as the brush. (**a**) 3M Scotch-Brite^TM^ Red (7447), (**b**) Grey (7448), (**c**) White (7445) and (**d**) SIFCO polyester jacket.

The rectifier used was a model pe2010 manufactured by Plating Electronic (Germany) following SIFCO’s specifications. The graphite anodes were covered by a cloth as a brush. Four different brush materials were used and structures are seen in high magnification images given in Fig. 2. Two of the materials contained abrasive particles and will be referred as abrasive cloths, while two that do not contain abrasive particles and will be called non-abrasive. Red Scotch-Brite^TM^ has abrasive particles embedded in the adhesive that holds the fibres together, while the abrasive particles in grey Scotch-Brite^TM^ are in the surface of both the adhesive and the fibres. On the other hand, white Scotch-Brite^TM^ has open fabric held together by an adhesive, and the polyester jacket has close-tied fibres and is much more compact than the others (see background of Fig. 2d).

Three parameters were selected as variables: the amount of particles suspended in the solution (bath load), the current density, and the cloth acting as the brush. Detailed values can be found in Table 2. Initial samples were electroplated with a bath load of 100 g/l, a current density of 37 A/dm^2^, and red Scotch-Brite^TM^ and will be considered as reference values. All other samples deposited share the values of two reference parameters, thus only one parameter is changed at any time, ensuring that any differences are due to one variable alone.

### Characterisation

The coating morphology was examined in an FEI XL30 ESEM and FEI XL30 SFEG scanning electron microscopes (SEM) by examining the surface and cross section of the samples using secondary electrons and back-scattered detectors. In all cases, images were taken with 20 kV at a working distance of 11 mm. An Olympus Lext OLS3100 confocal scanning laser microscope was used to map the surface of the coatings in three dimensions and roughness quantified using the R_a_ parameter. Ten random lines were averaged from an area of 1280 × 960 μm. X-ray diffraction (XRD) spectra were taken with a Philips D5005 diffractometer using Cu Kα radiation at 20 kV to determine the crystallographic structure of the coating. The Scherrer equation (equation (2)) is applied to calculate the mean crystallite size.


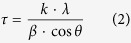


where *τ* is the main crystallite size in nm, *k* is the shape factor (0.9), *λ* is the X-ray wavelength (for Cu Kα = 0.15418 nm), *β* is the full width at half maximum in radians, and *θ* is the Bragg angle.

### Composition measurements

Composition was determined by energy dispersive x-ray spectroscopy (EDS) using Oxford Instruments Aztec software. An average of at least three measurements was taken from the surface (1547 × 1160 μm area) and additional measurements from coating cross sections. Discrepancies were found when comparing the results from the cross section with those taken from the surface, with higher aluminium content in many cases of the latter. The same trend is observed by another study of MMCs by Stroumbuli *et al*.^17^, although not explained. In this case, two facts may be contributing: the cross-sectional areas inspected are 22 times smaller than the surface measurements and some particles might be removed during cross section preparation. Consequently, surface measurements are used for the composition and are reported, while the cross-section measurements were used to determine if the particle deposition was homogeneous through the coating thickness.

## Results

### Optimisation of the particle flow

The original liquid-based design was tested against the adapted design at two different stirring speeds (350 and 700 rpm) to evaluate improvement in particle transportation. The results of the test are shown in Table 3. With the particle-based design, increased particle concentration and transportation is observed at all times in comparison with the liquid-based design by up to three times for minute 1 and nine times for minute 30. Slower stirring speeds work well with concentrated suspensions, whereas fast stirring is suited for dilute suspensions.

### The effect of bath load

Samples were produced with bath loads of 100, 250 and 500 g/l. Reference values were used for the other two parameters: current density (37 A/dm^2^) and fabric of the brush (red Scotch-Brite^TM^). All resulting samples have very similar characteristics in morphology, composition and roughness.

**Figure 3:**
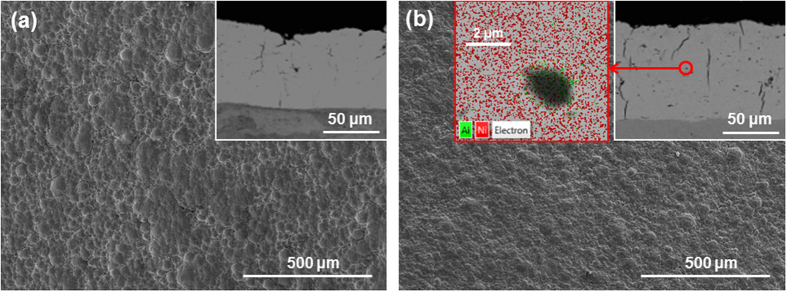
SEM images of samples produced with a current density of 37 A/dm^2^ and a brush of 3 M Scotch- Brite^TM^ Red (7447). (**a**) pure Ni coating, (**b**) Ni/Al coating (Bath load = 100 g/l). In both cases, the main image is taken from the surface with a secondary electron detector and the inserts are cross-sections taken with a back-scattered electron detector. The second insert in (**b**) is the superposition of a magnified back scattered image and EDS map, confirming dark particles shown in the cross-section inserts are Al.

No differences in morphology are observed for the selected bath loads compared to the reference coating. All resulting coatings are very compact and relatively smooth, while hinting at the usual nodular growth of the nickel matrix^18^,^19^,^20^. Representative images of the surface morphology and cross sections of the samples are compared against a pure nickel coating in Fig. 3. The morphology is the same for both Ni/Al and Ni coatings: the top of the nodules stand over a flat background and some cracking is revealed in the cross sections. The only difference between the coatings is the presence of Al particles in Fig. 3b, revealed by the back-scattered detector showing aluminium particles equally distributed thorough the thickness. Average roughness measurements confirm the similarity in morphology: R_a_ ranges between 3.8 ± 0.8 μm and 4.9 ± 0.7 μm and no correlation with bath load was found. Regarding coating composition, EDS analysis reveals a range of Al content between 1.4 ± 0.2 at.% and 7.6 ± 0.5 at.%, although values are not correlated with bath load.

**Figure 4:**
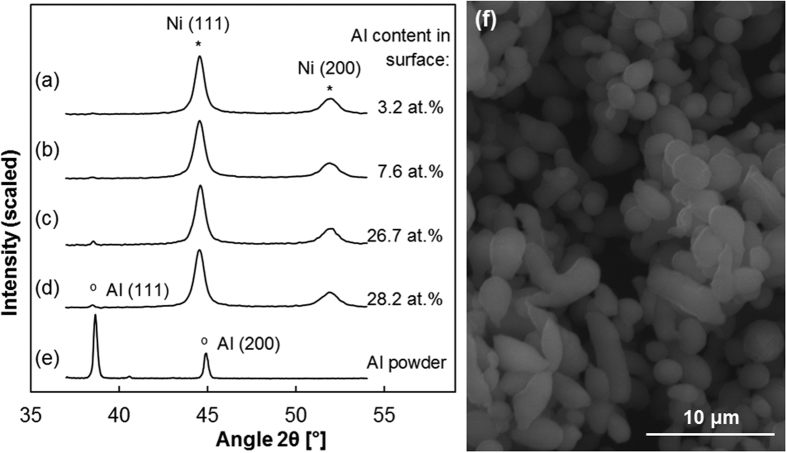
XRD patterns of different samples of Ni/Al coatings. (**a**) sample plated using reference values; for samples (**b–d**), the variable is (**b**) bath load = 500 g/l, (**c**) brush = white Scotch-Brite^TM^ (**d**) brush = polyester jacket. Finally, (**e**) corresponds to as-received Al powder particles, illustrated in (**f**) with a SEM image. Main peaks from a Ni FCC structure and Al FCC are highlighted, according to the PDF-2 International Centre for Diffraction Data (ICDD) database, (04–0850 Ni, 04–0787 Al).

X-ray diffraction spectra were taken to gather information about the crystalline structure. Two main peaks are clearly visible, which correspond to the FCC nickel matrix as in Fig. 4a. Samples with a bath load of 500 g/l hint at an additional peak at 38.5° (Fig. 4b), which corresponds to the more intense peak from diffraction of the Al powders (Fig. 4e). The diffraction spectra fit with a FCC aluminium microstructure. Presumably, the other coatings do not show this peak because of their lower aluminium content.

### The effect of current density

**Figure 5:**
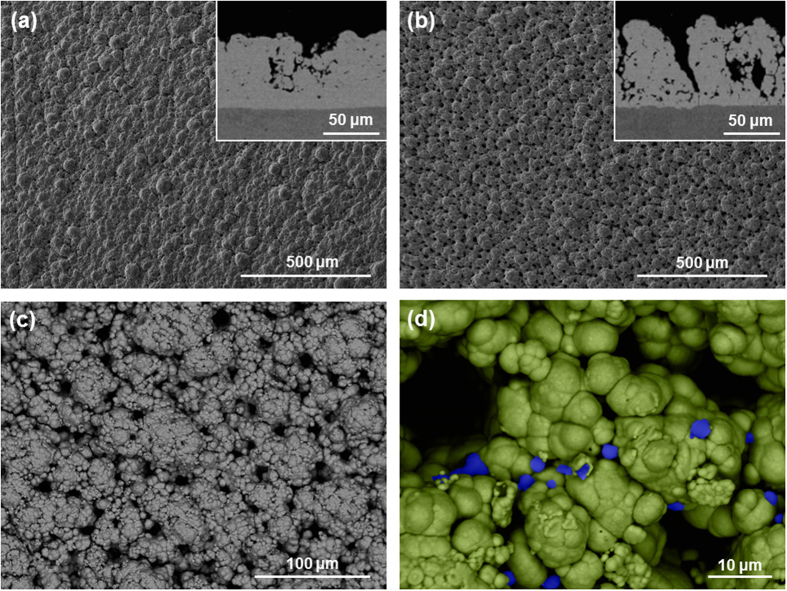
SEM images of samples produced with a bath load of 100 g/l, a brush of 3 M Scotch-Brite^TM^ Red (7447) and current densities: (**a**) 75 A/dm^2^ and (**b**) 124 A/dm^2^. In both cases, the main image is taken from the surface with a secondary electron detector while the inserts are cross-sections taken with a back-scattered electron detector. (**c**) and (**d**) are high magnification BSE images of (**b**). The small black dots in (**b**) and the false-coloured blue particles (**d**) are aluminium.

Samples were produced with current densities of 37, 75 and 124 A/dm^2^. Reference values were used for bath load (100 g/l) and brush fabric (red Scotch-Brite^TM^). Results show that the current density has an effect on the surface morphology and composition: as the current density increases, a rough, more accentuated globular structure is obtained, along with a higher number of incorporated particles. The samples produced with the lowest current density (37 A/dm^2^), described in the previous section, show a compact coating with relatively smooth globular growth (as Fig. 3b). The composition analysis of the samples with low current density show aluminium particles in low quantities (Table 4). On the other hand, samples plated with the highest current density (124 A/dm^2^) have a more marked globular structure, as illustrated in Fig. 5b. Deep and frequent pits make the coating less compact. High magnification observations reveal that the pits are pores, as they have a rounded shape (Fig. 5c). Large nodules visible at low magnifications (as in Fig. 5d) are formed by clusters of smaller nodules. Some aluminium particles are visible, trapped between boundaries of neighbouring nodules. The roughness and concentration of particles incorporated in these latter samples is much higher, as given in Table 4, and the distribution of particles is homogeneous thorough the cross-section. Finally, samples produced with medium current densities (75 A/dm^2^) seem to be at the threshold between both characteristics. Although the globular structure is more defined and some small pits are present (Fig. 5a), the overall morphology of the coating resembles more closely that with lower current densities. This is confirmed by both roughness and surface composition measurements (Table 4) reflecting similar values. Most probably, an intermediate state may be produced with current densities higher than 75 A/dm^2^, whereas current densities ranging from 37 to 75 A/dm^2^ produce very similar coatings. It is worth noting that the size of the nodules appears to be constant for all current densities (40–60 μm in diameter). There seems to be a relationship between roughness and aluminium content, as samples with comparable roughness have comparable amounts of aluminium as well.

### The effect of brush

**Figure 6:**
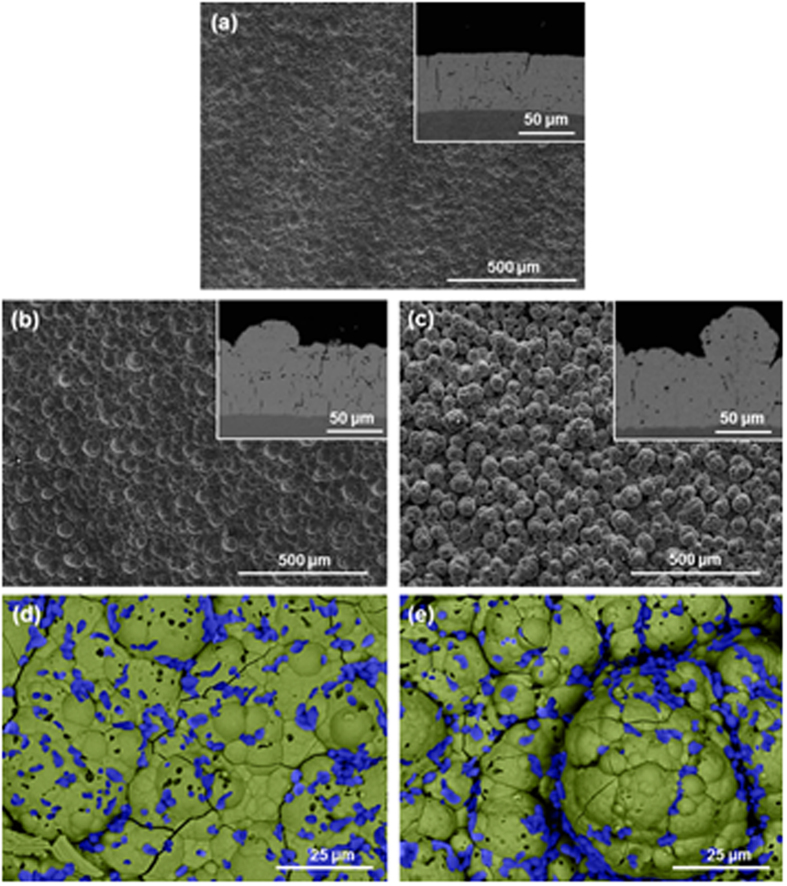
SEM images from the surface of samples produced with a bath load of 100 g/l, a current densities of 37 A/dm^2^ and different brushes: (**a**) grey Scotch-Brite^TM^, (**b**) polyester, and (**c**) white Scotch-Brite^TM^. The main image is taken with secondary electrons and the inserts are BS images of cross-sections, enabling visualization of aluminium particles (black dots). (**d**) and (**e**) are a higher magnification, back-scattered images of (**b**) and (**c**) respectively, false-coloured to show Al particles in blue and the Ni matrix in green.

Samples were produced with four brush materials: 3 M Scotch-Brite^TM^ red, grey and white and polyester jacket. Reference values were used for bath load (100 g/l) and current density (37 A/dm^2^). Both the coating morphology and the composition show variation depending on the fabric used. Images of the resulting deposited surfaces are shown in Fig. 6, and roughness and composition measurements are listed in Table 5.

The resulting coating using abrasive red Scotch-Brite^TM^ for the reference samples (described in the previous sections) is a compact, globular composite low in aluminium. Surface images of the coating using grey Scotch- Brite^TM^ abrasive fabric (Fig. 6a) show similarities with the morphology of samples plated with red Scotch-Brite^TM^. Roughness values and aluminium content seem to agree. The non-abrasive polyester jacket produces more accentuated globular structure than the abrasive cloths (Fig. 6b). Nodules appear to be smooth and more rounded in comparison with the latter. Roughness values confirm this as the R_a_ is higher with the non-abrasive brush material than for samples produced with abrasive cloths (Table 5). The aluminium content is also high for the polyester jacket coatings with 28.2 at.%. Microstructurally, XRD spectra show a Ni FCC structure with a small peak corresponding to Al FCC (Fig. 4c). Finally, the non-abrasive white Scotch-Brite^TM^ produces the most globular structure (Fig. 6c). In this case, the coating is less compact, although no rounded pits are present. The nodules appear to grow one on top of the other, with incomplete backfilling. Images show that that the bigger nodules are, in turn, formed by small nodules that cluster together, and confirmed by higher magnification images such as Fig. 6e. A number of aluminium particles are trapped in the crevices between nodules, as with the polyester jacket samples. Roughness and aluminium content are also high with a more stable composition around 26.7 at.% of aluminium for both samples. Roughness seems to be linked with particle deposition. Firstly, micrographs show that particles tend to accumulate in the border between nodules. Secondly, samples with a large concentration of particles exhibit increased roughness.

## Discussion

In this study, a traditional brush-plating setup was tested against a particle-optimised design. The results from section Optimisation of the particle flow highlight the importance of adapting the brush plating setup design to incorporate particles. Over time, the traditional setup underperformed because of the settling and precipitation of the particles: only 10% of the initial particles were circulated. By contrast, the particle-based design circulated around 90%. The changes undertaken kept a steady flow of the suspension by avoiding corners, gradients of velocity and areas where the liquid was stationary. This experiment highlights a potential problem when brush plating MMCs, while presenting a possible solution. To the best of our knowledge, this matter was only addressed for other variants of electroplating techniques^21^,^22^, thus no direct comparison can be made. In general, a good convection flux is recommended in order to keep the particles suspended and available to be captured in the coating, for instance by using agitation. This is in line with the sources consulted for solid-liquid flow^16^ and has been applied for the reservoir, although neither collection nor circulation of the suspension are contemplated due to the differences between brush and tank electroplating.

The total amount of Al particles incorporated seems to be slightly lower than research on MMCs using tank plating. In this current brush plating study, samples reached 28 at.% of aluminium particles, whereas a study of the same system (Ni/Al) using tank plating by Ghanbari & Mahboubi^8^ achieved 22 wt.% (equivalent to 38 at.%). This might be due to differences in the techniques, as the cloth might be brushing away some particles from the surface before they adhere. In any case, this occurrence does not seem to be heavily detrimental, as the results are similar in magnitude. The microstructure of the Ni matrix does not seem affected by the presence of Al particles neither in crystal orientation nor texture. Figure 4 shows in all cases very similar results of both the relative intensity of the second Ni peak (200) (ranging from 23.1% to 24.5% of the first Ni (111) peak) and the Full-Width at Half Maximum (FWHM) of the first and second peaks (0.63°–0.68° and 0.96°–1.10° respectively). Applying Equation 2 to the first Ni peak, the mean crystallite size range is between12.6–13.6 nm for the different samples, similar to the 14.4 nm obtained from the Ni/Al study from Ghanbari & Mahboubi^8^. Samples with over 20 at.% of Al show an Al (111) peak at 38.47° with a FWHM of 0.20°, which corresponds well with the spectra obtained with the as-received Al powder. Again, applying Equation 2 shows a mean crystallite size of 42.1 nm for the Al particles. The intensity of the peak is smaller than expected for the Al content measured with EDS, which can be explained by a combination of factors. Firstly, as particles are small (5 μm in diameter), a substantial part of the aluminium may oxidize in contact with air. Also, XRD penetration is deeper and accuracy is lower than EDS, thus readings may not match perfectly.

**Figure 7:**
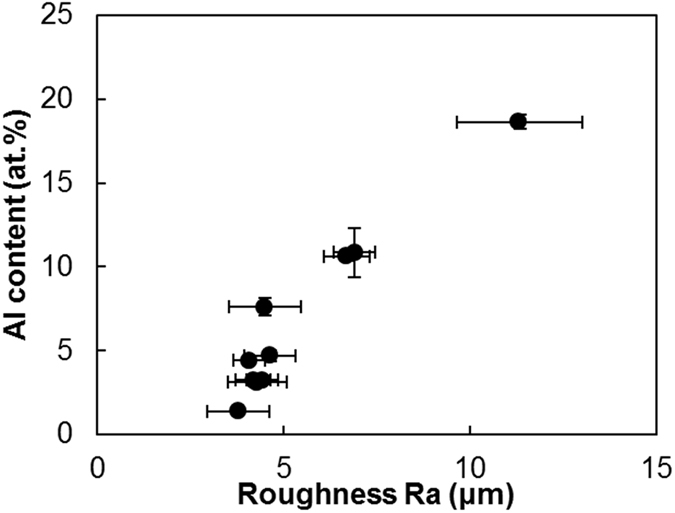
Aluminium content versus average surface roughness of the coatings produced with red Scotch- Brite^TM^ brush material.

**Figure 8:**
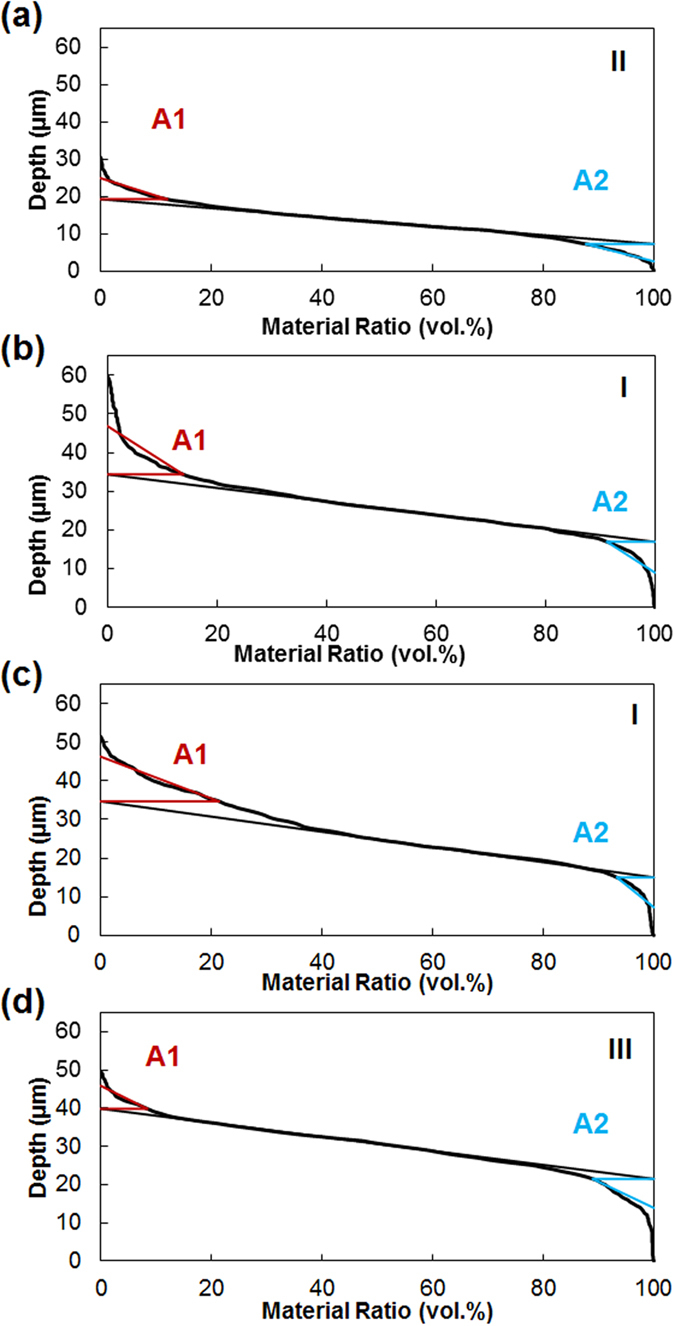
Abbott-Firestone curves of samples produced with a bath load of 100 g/l, a current density of 37 A/dm^2^ and different brush material: (**a**) red Scotch-Brite^TM^, (**b**) polyester and (**c**) white Scotch-Brite^TM^. (**d**) was produced with a bath load of 100 g/l, red Scotch-Brite^TM^ as the brush and a current density of 124 A/dm^2^. Roman numbers refer to the SZD.

The mechanism responsible for particle incorporation has been explained in earlier studies. Fransaer *et al*.^23^ identify both the forces that bring particles to the surface and the forces responsible for keeping the particles on the surface until they are engulfed by the matrix. The particles are mostly transported by means of convection to the sample surface. A particle remains on an electrode if the ratio of the normal to tangential forces acting on the particle is favourable. The tangential forces that work against the incorporation of particles are due to hydrodynamic shearing, whereas other tangential forces can promote the incorporation of particles. Fransaer *et al*. state that one such tangential force is the surface roughness of the particles. A mechanical bond is then developed between the two phases, with the particles being trapped by the growth of the metallic matrix^2^,^3^,^23^,^24^. Our observations show that regions between matrix nodules are more heavily populated by particles: these features seem to provide protection from the sweeping brush while serving as points for mechanical anchorage, where the particles remain until the matrix grows around them. Moreover, compositional measurements reveal that surface morphologies with abundant crevices tend to have higher aluminium content. Therefore, it seems that a rough surface can help to avoid excessive tangential forces that work against particle co-deposition. However, a quantitative measure of the abundance of crevices is needed to analyse its relationship with aluminium content. For this purpose, the use of average surface roughness R_a_ is proposed. Surface scans reveal that coatings with high R_a_ numbers correspond to coatings with more crevices and also match with coatings with higher aluminium content. The samples produced with red Scotch-Brite^TM^ illustrate the correlation between roughness and amount of particles incorporated in Fig. 7. Other traditional surface parameters have been considered, such as R_q_ (root mean squared of the profile) and 3D counterparts (S_a_ and S_q_), but results do not show any significant difference from R_a_. Ultimately, the roughness of the surface can be more accurately quantified by material ratio curves, or Abbott-Firestone curves. These curves are typically used to calculate the bearing area and give information about the abundancy and dimensions of peaks and valleys^25^,^26^, but they also quantify the relative abundance of peaks and valleys by giving the peak area (A1) and valley area (A2). In this study, the peak and valley areas determined are identical for samples produced with abrasive brushes (red and grey brushes), shown in Fig. 8a. Samples manufactured with non-abrasive brushes are characterised by peak areas (A1) four times larger than in the previous case. In Fig. 8b and c show that the shape of the curve is different for the white and polyester brushes, although both areas have the same value. Additionally, reduced peak height (R_pk_) and reduced valley height (R_vk_) are also larger than for samples produced with abrasive brushes, although the valley area (A2) has a very similar value in all cases. Finally, samples generated with high current densities show a valley area (A2) about 50% larger than samples generated with low current densities (Fig. 8d) and the peak area (A1) is about 25% smaller. In summary, Abbott-Firestone curves are able to quantify an abundant presence of peaks in samples of non-abrasive brushes and also the presence of pores for samples produced with high current density, as A1 and A2 are larger and smaller respectively. In both cases, the presence of peaks and valleys seem to provide protection from the sweeping brush and higher concentrations of particles are found.

Both the roughness-Al incorporation relationship and the use of R_a_ and Abbott-Firestone curves to characterise the surface may be applied beyond the Ni/Al system. Because of the mechanical nature of the bond between the particles and the matrix, this behaviour is possibly observed by a number of combinations of solid particles and metal matrices, as it should not be limited by chemical composition or chemical interactions between phases. This assumption would need future work to be evaluated, as it can help in creating coatings that hold a large number of particles.

**Figure 9:**
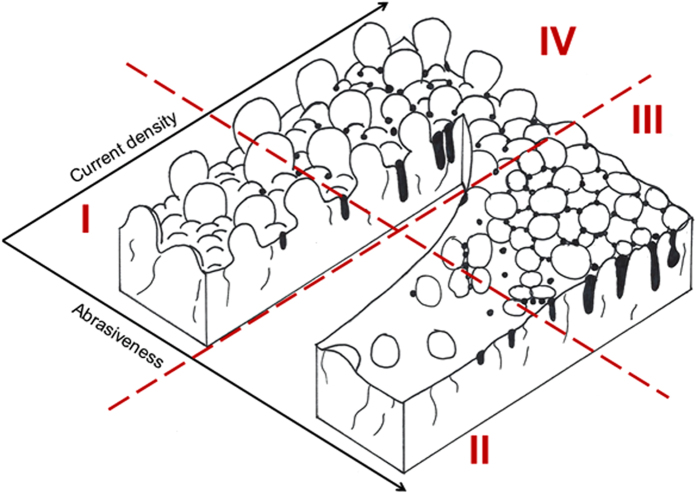
Structure Zone Diagram of Ni/Al coatings obtained with two key parameters: current density and abrasiveness of the brush. The presence of pores and globules distinguishes the structure of the coatings in four different regions.

Finally, the effect of current density and brush material on the coating can be illustrated using a Structure Zone Diagram (SZD) (Fig. 9). The SZD is divided into four regions depending on the appearance of two structural characteristics: pits and a defined globular structure. These pits are probably pores produced by evolving hydrogen: higher currents would imply larger quantities of gas being evolved, thus more pits; the circular shape would be produced by the hydrostatic pressures of the bubbles; and the rapid growth of the nodules would surround areas where bubbles are produced, making deeper pits. This effect is common knowledge for the electroplating community. On the other hand, the globular structure is more or less accentuated depending on the brush material. It is worth noting that to the best of our knowledge, the influence of the abrasiveness of the cloth has not been reported before, although it is common knowledge in the industry that using different brushes produces different results. Most likely, abrasive brushes grind the tips of the nodules as they grow, allowing valleys to reach the same height, creating new nucleation sites on the tips, producing smoother surfaces. Because the abrasion and the deposition take place simultaneously, the typical marks of abrasion may not show, as other small nodules grow immediately on top. On the other hand, coatings grown with non-abrasive cloths have a more defined globular structure, as shown in Fig. 6b and c, as the tops of nodules are not being ground. Both the appearance of pores and the globular structure have been quantified by the peak area (A1) and the valley area (A2), respectively, using Abbott-Firestone curves. Therefore, each region of the SZD has a characteristic Abbott-Firestone curve, as pointed out in Fig. 8. Region I shows an accentuated globular structure without pores, corresponding to the plot in Fig. 8b and c, produced by using non-abrasive clothes and low current densities. Coatings belonging to this region have the maximum particle content of all (27–28 at.%). Coatings from Region II are smooth, do not show pores (Fig. 8a), and are produced using abrasive clothes and low current densities. The particle content of coatings from this region is a minimum (between 1–8 at.%, although the average is 3–4 at.%). Region III is characterised by the presence of abundant pores and some globular structure, as the Abbott curve in Fig. 8d. Those coatings are produced with abrasive brushes and high current densities and have an intermediate particle content (11 at.%). None of the samples belong to Region IV, presumably they need to be produced by both a non-abrasive brush and high current density. Possibly, the surface would show a combination of abundant pores and also a marked globular structure such as Region I. However, confirmation of this hypothesis will be the basis of future work.

Additionally the effect of current density and bath load on particle incorporation was examined with abrasive cloths. Future work with non-abrasive brushes will be undertaken and also include the study of thermal stability of the final Ni/Al coatings, as well as formation of intermetallics through exothermic reactions.

## Conclusions

Brush plating of Ni/Al composite has been investigated in order to increase the Al particle content incorporated and a Structure Zone Diagram has been defined. Findings from particle transport show that sedimentation of particles can reduce particle transport to 10% in traditional setups. Continuous flow of the suspension was ensured by eliminating non-flowing areas. This improved particle transportation up to three times in short circulating periods and nine times in a longer run. Average surface roughness R_a_ has been used to evaluate the presence of crevices between nodules in the matrix. Coating surfaces with higher R_a_ values also report higher Al content and a link was established. Evaluation of several key deposition process parameters reveal that bath load has an impact on the stability of the process, but does not alter the surface morphology and Al incorporation, while both current density and brush material are influential on the coating morphology and the composition. Higher current densities and non-abrasive cloths produce a defined globular structure and high Al content, whereas low current densities and abrasive coatings produce smooth, compact coatings with lower particle content. A SZD was developed to illustrate the changes in surface structure and to help future developments of MMCs manufactured by brush plating: for instance, maximum particle content will be reached using the process parameters from region I; if a smooth surface is desired, the parameters selected should match those of region II. Future work in this area should include confirmation of the predictions for region IV, analysis of the effects of current density and bath load with non-abrasive cloths, and an in-depth study of the influence of abrasive particles on the growth of metal matrix composites.

## Additional Information

**How to cite this article:** Isern, L. *et al*. Structure zone diagram and particle incorporation of nickel brush plated composite coatings. *Sci. Rep.* **7**, 44561; doi: 10.1038/srep44561 (2017).

**Publisher's note:** Springer Nature remains neutral with regard to jurisdictional claims in published maps and institutional affiliations.

## Figures and Tables

**Table 1:** Solutions and plating conditions used in the brush plating process.

Step	Solution	Composition (weight %)	Conditions
Surface preparation	Electroclean	3% Sodium Citrate 2% Sodium Hydroxide	15 V
	Etching No. 2	11% Sodium Chloride 2% Hydrochloric Acid	9 V, reverse current
	Etching No. 3	15% Sodium Citrate 6% Citric Acid	15 V, reverse current
Pre-plating	Nickel Special	21% Nickel Sulfate 4% Citric Acid 2% Hydrochloric Acid 2% Acetic Acid	10 V, 2 μm thick
Plating	Nickel High Speed	11% Nickel Sulfate 10% Ammonium Formate 5% Ammonium Citrate 4% Ammonia Hydroxide	37–124 A/dm^2^

All products are commercially available under the name stated.

**Table 2:** List of variables and the corresponding values used to brush plate the samples to be studied.

Bath load (g/l)	Current density (A/dm^2^)	Brush
*100*	*37*	*3M Scotch-Brite*^*TM*^ *Red (7447*)^27^
250	75	3M Scotch-Brite^TM^ Grey (7448)^28^
500	124	3M Scotch-Brite^TM^ White (7445)^29^
—	—	SIFCO’s Polyester jacket^30^

In italics, values considered as base-line or reference.

**Table 3:** Measured concentrations of suspensions circulated through different electroplating setups for different times.

Setup	Original concentration = 20 g/l		Original concentration = 120 g/l	
	Concentration (g/l) at t = 1 min	Concentration (g/l) at t = 30 min	Concentration (g/l) at t = 1 min	Concentration (g/l) at t = 30 min
Liquid-based design	5	<1	72	13
Particle-based design (350 rpm)	14	12	101	108
Particle-based design (700 rpm)	18	27	92	98

**Table 4:** Results of average surface roughness R_a_ and Al content of the samples produced with different current densities, a bath load of 100 g/l and a brush of 3 M Scotch-Brite^TM^ Red (7447).

Current density (A/dm^2^)	Roughness R_a_ (μm)	Aluminium content (at.%)
37	4.2 ± 0.6	3.2 ± 0.1
75	4.4 ± 0.4	3.3 ± 0.2
124	6.8 ± 0.6	10.8 ± 1.2

The remaining composition is Ni.

**Table 5:** Average surface roughness R_a_ and Al content of samples produced with different brush material (further details in Fig. 2), a bath load of 100 g/l and a current density of 37 A/dm^2^.

Type of fabric on brush	Roughness R_a_ (μm)	Aluminium content (at.%)
Red	4.2 ± 0.6	3.2 ± 0.1
Grey	3.5 ± 0.4	3.9 ± 0.4
White	9.5 ± 1.6	26.7 ± 1.0
Polyester	6.5 ± 0.7	28.2 ± 0.5

The remaining composition is Ni.
